# Cerebral Calcifications as a Result of Primary Hyperparathyroidism: A Report of a Rare Case

**DOI:** 10.7759/cureus.57826

**Published:** 2024-04-08

**Authors:** Remzi R Hyusein, Stanimir Atsev, Atanas Boyukliev, Petar-Preslav Petrov, Vladislav Velchev, Plamen Penchev

**Affiliations:** 1 Faculty of Medicine, Medical University - Sofia, Sofia, BGR; 2 Cardiac Surgery Clinic, Passau Clinic, Passau, DEU; 3 Department of Endocrinology, University Multiprofile Hospital for Active Treatment "Kaspela", Plovdiv, BGR; 4 Department of Propedeutics of Internal Diseases, Medical University of Plovdiv, Plovdiv, BGR; 5 Department of Anatomy, Histology, and Embryology, Medical University of Plovdiv, Plovdiv, BGR; 6 Faculty of Medicine, Medical University of Plovdiv, Plovdiv, BGR

**Keywords:** parathyroid adenoma, primary hyperparathyroidism, parathyroid hormone, cerebral calcifications, case report

## Abstract

Primary hyperparathyroidism (PHPT) is an extremely uncommon cause of cerebral calcification. A male patient, aged 45, was admitted to the neurosurgery clinic with a closed traumatic brain injury, namely a concussion, resulting in symptoms of headache and loss of balance. A CT scan was conducted, which detected bilateral calcifications on the basal ganglia and the tentorium. The blood tests revealed increased levels of serum calcium, phosphate, and parathyroid hormone (PTH), while vitamin D levels were within the normal range. The patient received symptomatic therapy for the cerebral concussion and was referred for further diagnostic procedures. Based on these exams, it was determined that the patient had a parathyroid adenoma, which was responsible for PHPT characterised by increased levels of calcium, phosphate, and PTH. The patient subsequently underwent a successful parathyroidectomy. Half a year following the surgical procedure, the patient remained free of any indications of neurological conditions, and the levels of PTH and calcium in their body were within the expected range. Whenever trying to identify the cause of cerebral calcification, it is important to explore several possible diagnoses. A possible cause that should be taken into account is PHTP.

## Introduction

Less than 1% of cranial CT scans and MRI imaging reveal the presence of bilateral cerebral calcifications that impact the basal ganglia [1]. They have been observed in both cases without symptoms and in cases with various neurological disorders. Cerebral calcifications are categorised into three groups: physiological, idiopathic (including Fahr’s disease), and those that result from anomalies in calcium metabolism [2]. The predominant factors leading to these calcifications, which are dispersed in the striatum-pallidum-dentate, are conditions associated with calcium-phosphorus metabolism, including hypoparathyroidism, pseudohypoparathyroidism, pseudopseudohypoparathyroidism, and hyperparathyroidism. The correlation between basal ganglia calcifications and primary hyperparathyroidism (PHPT) is extremely rare [3].

PHPT is a prevalent hormonal condition characterised by abnormally high levels of calcium in the blood and raised or improperly normal amounts of parathyroid hormone (PTH) in the serum [4]. This phenomenon arises when one or more of the parathyroid glands overproduce PTH. In 85% of cases, the excessive production is caused by a parathyroid adenoma, in 10-15% of cases by four-gland hyperplasia, and in less than 1% of cases by parathyroid cancer [5,6].

The aim of this case report is to present a case study of a patient with a rare condition where cerebral calcifications were caused by PHPT and to contribute to the understanding and consideration of PHPT in cases of cerebral calcifications. 

## Case presentation

**Figure 1:**
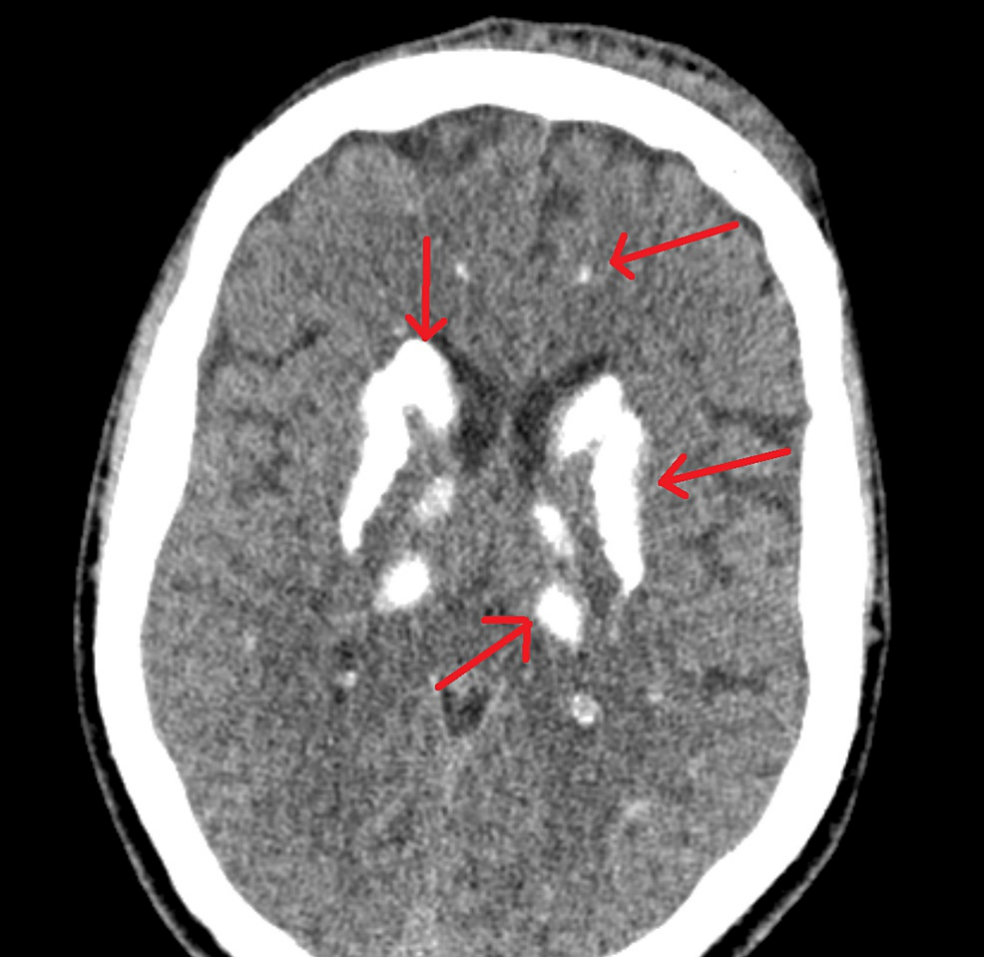
CT scan (axial plane). The arrows show calcifications on the putamen, globus pallidus, the tentorium itself and small calcifications in the frontal lobes and the thalamus.

A male patient, aged 45, presented to the neurosurgical department with a closed traumatic brain injury, specifically a concussion, accompanied by symptoms of headache and impaired balance. A CT scan was conducted, which identified calcifications in the basal ganglia (particularly the putamen and globus pallidus), on the tentorium itself, and small calcifications in the frontal lobes and thalamus (Figure 1).

**Figure 2:**
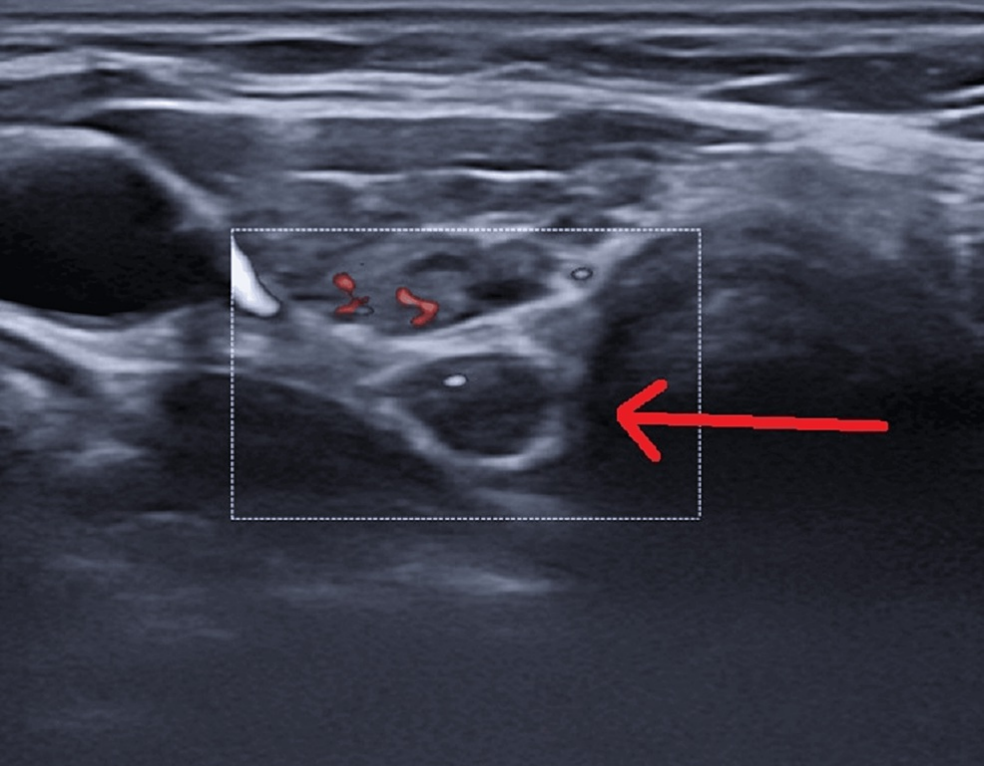
Ultrasound imaging. The arrow shows a parathyroid adenoma under the lower pole of the right thyroid lobe-hypoechoic formation with sharp and smooth outlines and increased blood flow.

The laboratory results showed high levels of calcium, phosphorus, and PTH, and normal levels of calcifediol (Table 1). The patient received symptomatic therapy for the cerebral concussion and was referred for further diagnostic tests. There have been no more neurological conditions noted in this patient following the treatment. The patient denied any familial occurrence of Fahr's disease, as well as any disorders related to calcium-phosphorus metabolism. The results of the parathyroid ultrasound imaging supported the expected diagnosis of PHPT caused by a solitary parathyroid adenoma located beneath the lower part of the right thyroid lobe. The adenoma appeared as a hypoechoic lesion with distinct and regular borders, and there was an observed increase in blood flow (Figure 2).

**Table 1:** Laboratory results. PTH: parathyroid hormone

Test name	Result	Reference range
Calcium	13.1 mg/dL	8.5-10.5 mg/dL
Phosphorus	4.8 mg/dL	2.5-4.5 mg/dL
PTH	165.2 pg/mL	11-67 pg/mL
Calcifediol	55 nmol/L	50-125 nmol/L

The patient then underwent a successful minimally invasive parathyroidectomy. The patient was mobilized on the day after the intervention. Surgery-related complications were not observed and the patient was discharged on the fifth day. Six months after the surgery, the patient still had no symptoms suggesting neurological problems, and PTH and calcium levels were within the normal ranges. The control CT scan showed no differences from the first one six months ago.

## Discussion

Recent studies indicate that the aetiology of PHPT is mostly unknown in the majority of patients. In all forms of PHPT, the pathophysiology involves the disruption of the normal mechanism that regulates blood calcium levels. This disruption is caused by an increase in the mass of parathyroid cells and/or a decrease in the number of CaSR proteins in these cells [4,7]. Consequently, elevated calcium levels are necessary to inhibit PTH levels. In our case, the diagnosis of PHPT was confirmed as all requisite criteria were satisfied, including the identification of a parathyroid adenoma and increased concentrations of serum calcium and PTH.

The biochemical characteristics of PHPT differentiate it from secondary and tertiary hyperparathyroidism. Secondary hyperparathyroidism is primarily caused by vitamin D deficiency, kidney disease, or hypercalciuria. It is characterised by an adequate increase in PTH levels in response to low calcium levels. Tertiary hyperparathyroidism is a medical disorder characterised by the independent activity of hyperplastic parathyroid glands, resulting in a state of hypercalcemia. This condition occurs as a consequence of protracted and severe secondary hyperparathyroidism. Walker and Silverberg reported that this phenomenon is predominantly encountered in individuals undergoing dialysis, although it can also manifest following a kidney transplant [4].

Postmenopausal women make up about 50% of the individuals affected by PHPT; nevertheless, the condition can develop in individuals of any age [4-6]. PHPT is frequently identified within the initial 10 years following menopause, in line with the recognised impact of oestrogen on the skeletal system. Oestrogen helps to counterbalance the excessive levels of PTH in the bones, which can lead to hypercalcemia.

Walker and Silverberg reported that conventional clinical signs of PHPT were mostly recognised prior to the 1970s and are now rare [4]. This condition involves a variety of symptoms that impact several systems in the body, including the skeletal, renal, gastrointestinal, neurological, and psychiatric systems [8,9]. Additional symptoms involve osteitis fibrosa cystica, nephrolithiasis, nephrocalcinosis, polyuria, polydipsia, anorexia, constipation, peptic ulcer disease, pancreatitis, muscle weakness, fatigue, and increased mortality [8,9].

At the moment, the majority of patients diagnosed with PHPT in the United States and Western Europe are categorised as asymptomatic, indicating that they are not experiencing the typical skeletal and renal symptoms associated with classical PHPT. Therefore, the majority of patients with PHPT are typically found by chance during normal laboratory tests that show elevated levels of calcium in the blood [4]. Additionally, we unexpectedly identified brain calcifications in our instance, encouraging us to perform additional examinations and eventually give the diagnosis of PHPT.

Only a small number of patients, specifically less than 10, who have both PHPT and cerebral calcifications, have been documented in the scientific literature [3,10-12]. The most often observed clinical signs are Parkinsonian symptoms, gait apraxia, frontal lobe dysfunction, and decreasing memory [3,10-12]. Additional symptoms that have been documented include a gradual decrease in emotional and intellectual functioning, changes in behaviour, seizures, epileptic episodes, stiffness in the muscles outside of the main body, slowed movement, impaired vision on the left side, and impaired movement and sensation in the left upper limb [3,11]. Our patient's lack of symptoms and absence of neurological problems is interesting. The reported calcifications are specifically located in the frontal and parietal lobes, dentate nuclei of the cerebellum, thalamus, caudate nucleus, globus pallidus, and basal ganglia [3,10-12]. These findings align with those seen in the previously cited cases.

Hypoparathyroidism and Fahr's disease are common causes of basal ganglia calcifications [3]. The term "Fahr's disease" originated in memory of Karl Theodor Fahr, a German neurologist who initially documented this medical condition. As stated by Saleem et al., this is an uncommon neurological disorder characterised by the spontaneous calcification of the basal ganglia. It is frequently inherited in an autosomal dominant manner [13]. It has been determined that neither of those two prerequisites existed in this situation.

## Conclusions

This study examines a patient with an extremely rare condition known as cerebral calcifications, which was caused by PHPT. Furthermore, it is noteworthy that our patient was asymptomatic and did not present any neurological, renal, or musculoskeletal complications. Unlike earlier reports of this condition, which typically include neurological abnormalities, this study is unique in that it does not reveal any such abnormalities. This distinguishing feature differentiates it from other reports on the same condition. There are other potential causes for the development of cerebral calcifications, and this case report adds to the evidence supporting the inclusion of PHPT as one of these causes. Physicians should be aware that cerebral calcifications can sometimes occur as a result of PHPT and may mimic Fahr's disease.
